# Supplementary figures and images for: Clinical Benefits of Exogenous Ketosis in Adults with Disease: A Systematic Review
Source: Nutrients. 2025 Sep 30;17(19):3125. doi: 10.3390/nu17193125 (PMC12525594; doi:10.3390/nu17193125)

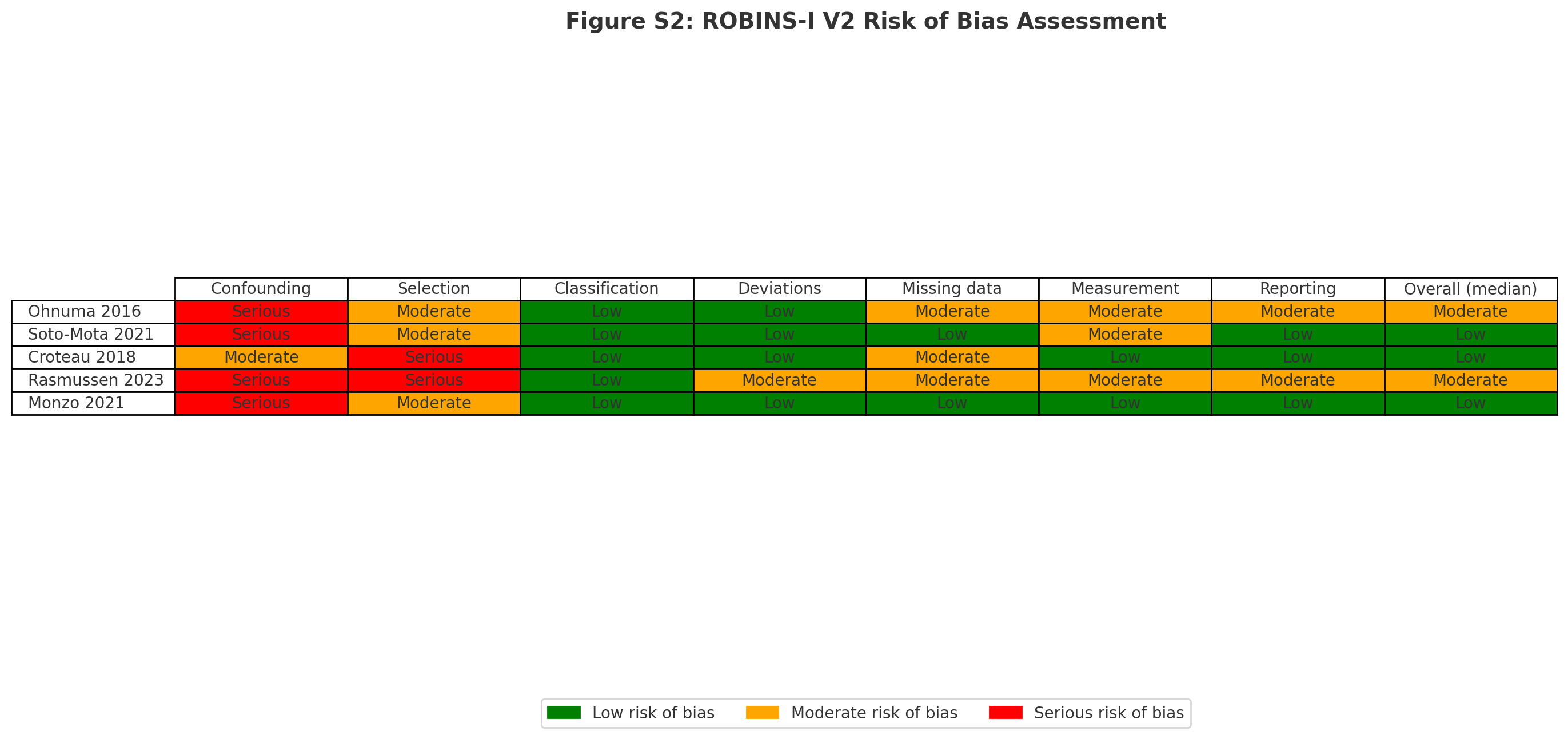

Supplement: Supplementary file 1 [file nutrients-17-03125-s001.zip › Supplementary Material 2.png]
